# Complete genome sequence of the environmental *Pseudomonas mediterranea* strain Cas656 isolated from rhizosphere soil in China

**DOI:** 10.1128/mra.00348-25

**Published:** 2025-06-12

**Authors:** Hongquan Sun, Fuliang Chen, Youai Zhang, Xiaoxiang Chen, Weimin Wang, Qingwei Cui, Yongfeng Ai, Guanglong Song, Jinchang Liang, Yonghua Chen, Xiaoqiang Wang

**Affiliations:** 1 Tongren Tobacco Science and Technology Center, Tongren, Guizhou, China; 2 China Tobacco Zhejiang Industrial Science and Technology Center 561889 , Hangzhou, China; 3 Key Laboratory of Tobacco Pest Monitoring & Integrated Management, Tobacco Research Institute of Chinese Academy of Agricultural Sciences 243822 , Qingdao, China; The University of Arizona, Tucson, Arizona, USA

**Keywords:** complete genome, *Pseudomonas mediterranea*, rhizosphere soil

## Abstract

A bacterial strain *Pseudomonas mediterranea* Cas656 was isolated from the rhizosphere soils of tobacco in China. Here, we present the complete genome sequence of *P. mediterranea* Cas656. The genome comprises a 6,245,034 bp circular chromosome, with G + C contents of 60.92%.

## ANNOUNCEMENT

*Pseudomonas mediterranea* belongs to the genus *Pseudomonas*, which is characterized by a highly diverse and metabolically versatile (1, 2). To get insights into the genome features of *P. mediterranea*, we isolated and sequenced its genome. Microbial samples were collected from rhizosphere soils in a tobacco-producing field in Xuan’en country (29°59′1.932″N, 109°35′2.976″E), China, on 15 August 2023. The soil samples were serially diluted and subsequently plated on nutrient broth agar (NA) plates. After 48 h at 28°C of cultures, the single colonies were picked up and purified three times on NA plates. The species were identified by PCR and Sanger sequencing of 16S rRNA genes (accession number PV082957) using the primers 27F/1492R (3). The greatest sequence similarity was 99.79% to the type strain of *P. mediterranea* CFBP 5447^T^ (accession number AUPB01000004).

The single colony was picked up from NA plates and incubated into 100 mL nutrient broth medium for 24 h (28°C, 200 rpm). The genomic DNA of strain Cas656 was extracted by phenol/chloroform extraction (4). DNA purity and contents were detected by agarose gel electrophoresis and a Qubit, respectively. The same DNA extractions were used for PacBio and Illumina libraries. The PacBio libraries were constructed by SMRTbell Express Template Prep Kit 2.0 (Pacific Biosciences, Menlo Park, CA) and then sequenced on a PacBio platform (20 kb inserts library). The Illumina libraries were prepared by NEB Next Ultra DNA Library Prep Kit (New England Biolabs, USA) and sequenced on the Illumina PE150 platform (270 bp inserts library; Illumina, San Diego, CA). The low-quality PacBio reads were filtered by the SMRT Link (v8.0) and generated 14.88 Mbp and 117,180 reads with the N50 read length of 16,336 bp. Illumina reads with a quality score <15 accounting for more than 40% of the total number of reads were filtered by fastp (5) and generated 1,639 Mbp clean data. The high-quality PacBio sequence of Cas656 was assembled using Canu v2.0 (6) and cyclized using Circlator v1.5.5 (7) by overlapping sequence ends. The contigs were polished using Pilon 1.16 (8) according to the Illumina clean data. Finally, the misassemblies of the genome were detected using PEARP and corrected manually (9). Default parameters for all software applications were used in genome assembly unless otherwise specified.

The assembled genome of Cas656 consists of one contig without gaps with 6,245,034 bp in length. The genome has a G + C content of 60.92% (Table 1). The genome was annotated by Prokaryotic Genome Annotation System (Prokka) v1.12 (10), revealing 5,482 coding genes. tRNA-encoding genes and rRNA operons were predicted by using tRNAscan v 1.3.1 (11) and RNAmmer v1.2 (12), respectively. The genome has 66 tRNA and 5 16S rRNA.

**TABLE 1:** Summary of genome information

Parameters	Value
Number of contigs	1
Genome sizes	6,245,034 bp
G + C content	60.92%
Circular	Yes
Number of coding sequences	5,482
Gene total length	5,475,207 bp
Gene length/genome	87.67%
Number of tRNA genes	66
Number of 5S rRNA genes	6
Number of 16S rRNA genes	5
Number of 23S rRNA genes	5

## Data Availability

This Whole Genome Shotgun project has been deposited at GenBank under accession CP186024. The version described in this paper is version CP186024.1. The raw sequencing reads generated from the PacBio and Illumina platforms have been submitted to SRA, and the corresponding accession numbers are SRR32919522 and SRR32917559, respectively.
